# Patient-reported pain and hand function: important determinants of the postoperative results one year after PIP arthroplasty

**DOI:** 10.1016/j.jham.2026.100421

**Published:** 2026-01-14

**Authors:** Bo J.W. Notermans, Joris S. Teunissen, Ruud W. Selles, Luitzen H.L. de Boer, Brigitte E.P.A. van der Heijden, the Hand-Wrist Study Group∗

**Affiliations:** a Department of Plastic Surgery, Reconstructive and Hand Surgery, Radboudumc, Nijmegen, the Netherlands; b Department of Plastic, Reconstructive and Hand Surgery, Erasmus MC, Rotterdam, the Netherlands; c Department of Rehabilitation Medicine, Erasmus MC, Rotterdam, the Netherlands; d Hand and Wrist Centre, Xpert Clinic, the Netherlands; e Department of Plastic Surgery, Jeroen Bosch Ziekenhuis; 's-Hertogenbosch, the Netherlands

**Keywords:** Arthroplasty, Osteoarthritis, PROMs, Hand surgery, Michigan hand outcomes questionnaire

## Abstract

**Aim:**

This study aimed to assess determinants associated with patient-reported pain and hand function 12 months after proximal interphalangeal joint arthroplasty for osteoarthritis.

**Methods:**

Prospectively gathered data of 113 patients, who completed the Michigan Hand outcomes Questionnaire preoperatively and 12 months postoperatively, was used. We compared pre- and 12-month postoperative (categorized) MHQ pain and hand function scores and assessed determinants associated with the postoperative scores.

**Results:**

The Michigan Hand outcomes Questionnaire pain and hand function scores at intake were 41 (SD 19) and 52 (SD 17), respectively, and 65 (SD 23) and 63 (17) after surgery. Less pain at intake was associated with less pain 12 months postoperatively. The following determinants were associated with better hand function after surgery: better hand function at intake, surgery on the dominant hand, and the use of surface replacement implants.

**Conclusion:**

MHQ scores at intake were significantly associated with postoperative MHQ scores. This knowledge can be used during preoperative consultation to improve shared decision making and create adequate patients’ expectations.

**Level of evidence:**

III

## 1 Introduction

Patients with symptomatic proximal interphalangeal (PIP) osteoarthritis, not responding to conservative treatment, can be treated with arthroplasty. Pain has been reported to be the main indication for surgery. 1, 2, 3, 4, 5, 6, 7 Many previous reported studies describe an adequate pain release after implantation of different types of PIP joint prostheses for different indications and diagnoses.^1^^,^^4^^,^^7^, ^8^, ^9^, ^10^, ^11^, ^12^, ^13^, ^14^.

The second most common complaint of patients with PIP osteoarthritis, which is in many cases associated with pain, is the loss of motion and deterioration of hand function.^3^^,^^7^^,^^15^ Regarding the range of motion after PIP arthroplasty, widely divergent results have been reported. An overview of functional outcomes by implant type and surgical approach was previously published by Yamamoto et al.^16^

The Michigan Hand outcomes Questionnaire (MHQ) is a validated questionnaire with the ability to measure, among other things, both the patient-reported pain and hand function.^17^ Previous studies report a reduction of patient-reported pain and maintenance of hand function after PIP arthroplasty, measured using the MHQ.^1^^,^^4^ In line with this, in an earlier study, we found a significant reduction of MHQ-pain score 12 months after PIP arthroplasty among 98 patients.^18^ In 60 percent of patients, this pain reduction was perceived as clinically relevant. The other MHQ domains also improved significantly. For all outcome domains, however, we noticed a large between-subject variability in the improvement at follow-up. This raised the question of which determinants might be associated with patient-reported outcomes, in particular pain and hand function, and whether there is a group of patients that does not improve or even get worse after surgery. Ono et al. have previously described that patients without complications have a significantly better MHQ-pain score postoperatively, but other possible determinants affecting postoperative patient-reported pain and hand function remain unclear.^4^

Therefore, the primary aim of this study (including the former group of 98 patients) was to assess which determinants are associated with patient-reported pain and hand function 12 months after proximal interphalangeal joint arthroplasty for osteoarthritis.

Knowledge of patient- and surgery-specific factors that are associated with patient-reported pain and hand function are of great value to optimise preoperative patients' information and create adequate expectations.

## 2 Patients and methods

### 2.1 Patients

This study describes prospectively gathered data of a consecutive, population-based sample of patients treated in daily hand surgery practice,^19^ reported following the STROBE statement.^20^ Patients included in this study underwent a PIP arthroplasty for either primary degenerative or posttraumatic osteoarthritis between June 2009 and July 2019. As described in an earlier study,^18^ patients were considered for surgery if they had radiological signs of PIP osteoarthritis (Kellgren Lawrence classification ≥ grade 2) in combination with pain, despite nonsurgical treatment for at least three months.^21^ Besides pain, stiffness and/or deformity were indications for surgery. Exclusion criteria consisted of age <18 years at the time of surgery, corticosteroid injection within three months before surgery, and patients with inflammatory arthritis. After written consent during the first consultation, patients were invited to complete electronic questionnaires preoperatively. We only included patients if they had completed the MHQ preoperatively and 12 months after surgery.

### 2.2 Surgical procedure

Surgery was performed by 20 surgeons, who were all hand fellowship-trained with a level of experience ranging from 3 to 5 according to the classification by Tang and Giddins.^22^ Surgery was performed under local or plexus anaesthesia in a bloodless field (with an arm or finger tourniquet). In most cases (n = 111), a dorsal longitudinal skin incision was made over the PIP joint and the central tendon was split longitudinally (n = 94) or according to Chamay (n = 17) to expose the joint.^23^ One joint was already exposed laterally due to trauma and one joint was replaced using a volar approach. After preparing the joint, a trial prosthesis was introduced. Function and stability were tested and a permanent implant was chosen; silicone (NeuFlex® DePuy Orthopaedics, Warsaw, Indiana, USA) or SR (Avanta ® Avanta Orthopaedics, San Diego, USA until 2014 and Stryker ® Stryker, Kalamazoo, MI, USA). The selection of the implant was based on surgeon preference and experience and was made preoperatively. Apart from the difference in the prosthesis related implantation set used, there were no surgical technical differences between the two implants. Position and alignment were checked by X-ray in two positions (PA and lateral). The extensor tendon was repaired with absorbable sutures. The skin was closed using nonabsorbable sutures and an extension splint is applied.

### 2.3 Postoperative treatment

The cast was removed after 3–5 days and a static extension splint in neutral PIP joint position was made by a hand therapist. If hyperextension was present the splint was adjusted adding an extension block of 10–30°, depending on the severity of the hyperextension. Physical therapy was initiated, starting with active exercise therapy without resistance during the first 8 weeks. During therapy, the static splint was removed and flexion was practiced over a pre-bent splint, whereby the number of degrees of flexion was gradually built up during the weeks to the maximum possible. The use of the extension splint was reduced during the day after 7–8 weeks but continued at night. After 8 weeks, exercises with resistance were build up slowly. After 3 months the use of the splint at night was reduced. If indicated, therapy was continued, until 6–12 months after surgery. Three months after surgery a follow-up visit with the surgeon was scheduled and, if indicated, an X-ray was obtained.

### 2.4 Data collection

Data collection was part of routine outcome measurement using GemsTracker electronic data capture. GemsTracker^24^ (Generic Medical Survey Tracker) is a secure web-based application for the distribution of questionnaires and forms during clinical research and quality registrations; details have been published earlier.^19^ The local Medical Research Ethical Committee approved our study. All patients provided consent for their data to be used in this study.

The Michigan Hand outcomes Questionnaire^17^ is a hand-specific patient-reported outcome measurement, which contains six subscales; (1) overall hand function, (2) activities of daily living, (3) pain, (4) work performance, (5) aesthetics, and (6) patient satisfaction with hand function. All 57 questions are answered on a five-point Likert scale. We used the pain and overall hand function subscales, of which outcome values range from 0 to 100. Zero indicates poor hand function and a lot of pain, 100 indicates excellent function and no pain.

We evaluated preoperative and postoperative MHQ pain and hand function scores and evaluated the change in categorized MHQ scores; (1) 0–25, (2) 26–50, (3) 51–75, and (4) 76–100. Our primary outcome was to assess which factors are associated with the patient-reported pain and hand function, both measured using the MHQ 12 months postoperatively. The explained variance of the outcome on the MHQ pain and hand function scale was determined. Factors evaluated for their possible association with our outcomes were: age, sex, diagnosis, type of implant, operation of the dominant hand, an operation on multiple fingers, complications (including a reoperation of the initially operated finger within 12 months after the initial surgery and an antibiotically or surgically treated infection), hand comorbidity (including polyarthritis - meaning degenerative arthritis in multiple fingers or joints -, carpal tunnel syndrome, stenosing tenovaginitis, and Dupuytren's disease) duration of symptoms, work status and, depending on the model, MHQ pain or MHQ hand function score at baseline.

### 2.5 Statistical methods

Medians and interquartile ranges are reported for non-normal distributed data, means with 95 % confidence intervals are reported for normally distributed data. Normality was assessed by plotting histograms. We performed a non-responder analysis of the whole group to test if missingness was dependent on any recorded feature. Patients who completed the MHQ were called responders, whereas a non-responder was defined as a patient who did not complete the postoperative questionnaire (Table S1). We used the Chi-squared test to compare categorical data, T-tests for normal distributed numerical data, and Wilcoxon Rank-Sum tests for non-normal distributed numerical data. To evaluate the difference in MHQ scores we used a paired *t*-test. Based on clinical knowledge and literature, the authors agreed upon a selection of variables that were expected to be possibly associated with patient-reported pain and function. A maximum of variables was set based on the study sample (n = 11).^25^ All variables were simultaneously added to the multivariable linear regression models. The R^2^ was assessed for the model's explained variance. A P-value smaller than 0.05 was considered statistically significant. Clinical relevance was assessed using the Minimal Clinically Important Difference (MCID), set at 10 and 24 points on the MHQ hand function and pain score, respectively.^26^

## 3 Results

Between January 1st, 2009 and July 1st, 2019, 184 patients underwent a PIP arthroplasty. We excluded three patients who were diagnosed with inflammatory arthritis and 68 patients who did not complete the MHQ questionnaire twelve months after surgery. Therefore, 113 patients were included for analysis. Besides age (63 (SD 9.3) versus 59 (SD 11), *p* = 0.029), no significant differences were seen in baseline variables between responders and non-responders (Table S1).

**Supplementary Fig. 1:**
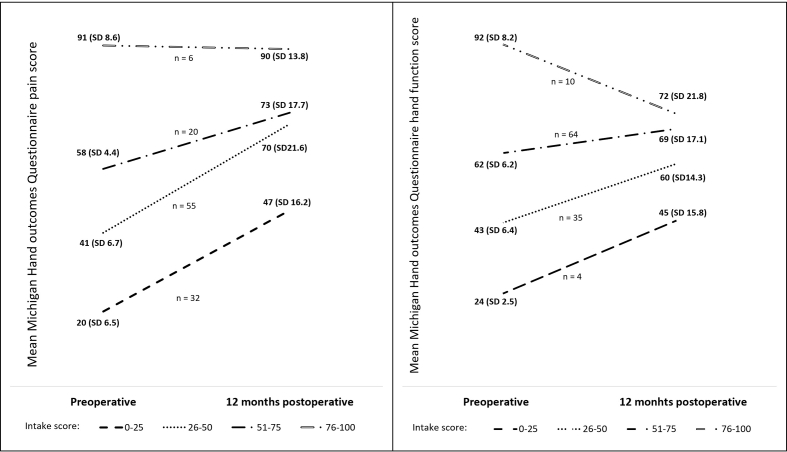
Pre- and postoperative Michigan Hand outcomes Questionnaire pain and hand function scores.

Baseline characteristics of the study sample and surgery specific variables are shown in Table 1. The mean baseline MHQ pain score of 41 (SD 19) increased to 65 (SD 23, *p* < 0.001) at 12 months, and the mean baseline MHQ hand function score of 52 (SD 17) increased to 63 (SD 17) at 12 months after surgery (*p* = 0.016). The postoperative MHQ pain and hand function scores when categorized into four groups based on intake score to provide a visualization of the linear variable used in the model and are presented in Supplementary Fig. 1.

**Table 1:** Patient characteristics.

Total amount of patients, n = 113	
Variables	
Age, mean (SD)	63 (9.3)
Sex	
Male	27
Female	86
Smoking	
Yes	12
No	74
Unknown	27
Diabetes	
Yes	6
No	53
Unknown	54
Operated on dominant hand	
Yes	60
No	53
Diagnosis	
Primary degenerative	87
Post-traumatic	26
Duration of symptoms	
(months), median [IQR]	24 [12–60]
Work status	
No work	67
Light physical work	20
Medium physical work	20
Heavy physical work	6
Type implant	
Silicone	35
Surface replacement	78
Hand comorbidity	
Yes	60
No	53
Operated on	
One finger	92
Two fingers	21
Complication	
Yes	17
No	96

The multivariable model to assess factors associated with the MHQ pain score 12 months after surgery is displayed in Table 2. The explained variance in the outcome of this model was 29 %.

**Table 2:** Multivariable model MHQ^1^ pain score 12 months postoperatively.

Determinant		beta	95 % Confidence interval	P-value
Age (per year)		0.074	−0.47; 0.62	0.79
Sex	Female	*ref*^b^		
	Male	7.4	−2.1; 17	0.13
Diagnosis	Primary degenerative	*ref*^b^		
	Posttraumatic	−1.9	−13; 9.1	0.74
Implant type	Silicone	*ref*^b^		
	Surface replacement	−4.1	−13; 4.7	0.36
Operated on	Non-dominant hand	*ref*^b^		
	Dominant hand	4.8	−3.2; 13	0.24
Operated on	One finger	*ref*^b^		
	Two fingers	6.4	−4.6; 17	0.25
Hand comorbidity	No	*ref*^b^		
	Yes	−6.2	−15; 2.9	0.18
Work status	No (paid) work	*ref*^b^		
	Light physical work	−1.2	−13; 10	0.84
	Medium physical work	−0.59	−12; 11	0.92
	Heavy physical work	4.7	−14; 24	0.63
Duration of symptoms (per month)		0.015	−0.020; 0.049	0.40
Complication	No	*ref*^b^		
	Yes	−6.2	−17; 5.1	0.28
MHQ^a^ pain intake		0.44	0.21; 0.67	*0.000*

R^2^ = 0.29.

a MHQ = Michigan Hand outcomes Questionnaire.

b *ref* = reference, beta = 1.0.

The multivariable model to assess factors associated with the MHQ hand function score 12 months after surgery is displayed in Table 3. The explained variance of the outcome of this model was 30 %.

**Table 3:** Multivariable model MHQ^1^ hand function 12 months postoperatively.

Determinant		beta	95 % Confidence interval	P-value
Age (per year)		0.15	−0.25; 0.54	0.47
Sex	Female	*ref*^b^		
	Male	5.7	−1.2; 13	0.11
Diagnosis	Primary degenerative	*ref*^b^		
	Posttraumatic	3.4	−4.7; 12	0.41
Implant type	Silicone	*ref*^b^		
	Surface replacement	6.7	0.24; 13	*0.042*
Operated on	Non-dominant hand	*ref*^b^		
	Dominant hand	6.3	0.50; 12	*0.034*
Operated on	One finger	*ref*^b^		
	Two fingers	0.53	−7.3; 8.7	0.90
Hand comorbidity	No	*ref*^b^		
	Yes	−4.0	−11; 2.6	0.23
Work status	No (paid) work	*ref*^b^		
	Light physical work	2.3	−6.1; 11	0.59
	Medium physical work	4.8	−3.7; 13	0.26
	Heavy physical work	13	−1.5; 27	0.079
Duration of symptoms (per month)		0.0062	−0.019; 0.031	0.63
Complication	No	*ref*^b^		
	Yes	−0.59	−8.7; 7.6	0.89
MHQ^a^ function intake		0.29	0.11; 0.47	*0.002*

R^2^ = 0.30.

a MHQ = Michigan Hand outcomes Questionnaire.

b *ref* = reference, beta = 1.0.

## 4 Discussion

This study aimed to identify patient- and surgery-related factors associated with patient-reported pain and hand function 12 months after PIP joint arthroplasty. We found that baseline scores were the strongest predictors: patients with less preoperative pain reported less pain postoperatively, and those with better baseline hand function reported better hand function at 12 months. In addition, surgery on the dominant hand and the use of surface replacement implants were associated with slightly higher postoperative function scores, although these differences did not exceed the Minimal Clinically Important Difference (MCID) of 10 points and should therefore be interpreted as not clinically meaningful at the group level.

Our findings are consistent with our earlier study of this cohort, in which we reported significant improvements in MHQ pain and function scores at one year and higher hand function scores in patients receiving SR implants compared with silicone implants.^18^ Although these associations were statistically significant, the magnitude of improvement (6.3–6.7 points) fell below the established MCID for hand function.^26^ Murray et al. also described the possible stifness and limited range of motion postoperatively, which is generally tolerated when pain has been eliminated.^27^

Recent studies reporting PROMS after PIP arthroplasty typically focus on postoperative scores and complication rates, but do not examine determinants of patient-reported outcomes. Meuser et al. reported excellent brief MHQ and VAS pain scores after SR arthroplasty, with low revision rates and improvement of axial deviation.^28^ Reischenböck et al. similarly found sustained improvement in PROMs and radiographic alignment at 5 years,^29^ while Riggs et al. demonstrated significant MHQ improvements after volar-approach silicone arthroplasty.^30^ Our study adds to this literature by evaluating associations between baseline factors, complications, and postoperative PROMs. We found no association between complications requiring reoperation and 12-month MHQ pain or function scores.

Although limited, earlier studies have reported postoperative MHQ results similar to ours, regardless of whether silicone or SR implants were used. Namdari et al. reported somewhat higher MHQ scores across domains, but their cohort included only silicone implants.^31^ Amirtharajah et al. reported similar results in a small cohort of SR implants.^32^ Subjective, as well as objective functional outcomes, such as range of motion, remain inconsistently reported. These inconsistent findings underscore the need for longitudinal patient-reported analyses and better integration of objective and subjective outcomes over time.

We observed significant improvements in pain and hand function from baseline to 12 months. However, because the MCID for MHQ pain is 24 points,^26^ patients presenting with baseline pain scores above 75 may experience limited clinically relevant pain reduction in the absence of additional surgical indications. When surgery is performed for other reasons, preoperative counseling should include clear communication about the expected magnitude of symptom improvement.

Surgery on the dominant hand was associated with higher postoperative hand function scores. Although this has not been described previously in the context of PIP arthroplasty, Burke et al. reported similar findings for MHQ work and ADL domains after trapeziometacarpal arthroplasty.^33^ A plausible explanation is that patients rely more heavily on their dominant hand and may therefore perceive postoperative improvements more strongly.

A major strength of this study is that it evaluates patient- and surgery-related factors associated with standardized PROMs collected prospectively in routine hand surgery practice. However, several limitations should be considered. First, the sample size is relatively small for multivariable modelling, which may have reduced the ability to detect contributing factors that could become significant in a larger cohort. Second, not all patients completed the MHQ at 12 months despite reminders, introducing potential selection bias. This reflects our pragmatic design, in which PROMs are collected remotely to avoid additional burden for patients. Baseline characteristics did not differ between respondents and non-respondents, except for a modest age difference of four years, which is unlikely to be clinically meaningful. Finally, although baseline pain and hand function were significant predictors of postoperative scores, the models explained only 29–30 % of the variance. This suggests that additional factors, such as psychosocial variables, patient expectations, or perioperative care experience, may substantially influence patient-reported outcomes.^34^, ^35^, ^36^, ^37^, ^38^, ^39^ Future studies incorporating such variables, and including longer follow-up periods, are warranted to further refine prediction models and guide shared decision-making.

## Authors’ contributions

B.J.W.N., J.T., R.S. and E.P.A.H supported in study design. B.J.W.N. and J.T. performed data assembly and analysis. B.J.W.N., J.T., H.L.B., R.S, and E.P.A.H helped in initial draft and final approval of manuscript.

## Contributorship

BJW Notermans wrote the first draft of the manuscript. All authors reviewed and edited the manuscript and approved the final version of the manuscript.

## Informed consent

Patients provided written consent for the utilisation of their data for clinical research.

## Ethical approval

The Medical Research Ethics Committee Erasmus MC approved this study.

## Funding

None.

## Financial disclosure statement

The author(s) received no financial support for the research, authorship, and/or publication of this article. None of the authors has a financial interest in any of the products, devices, or drugs mentioned in this manuscript.

## Declaration of competing interest

The authors declare that they have no known competing financial interests or personal relationships that could have appeared to influence the work reported in this paper.
