# Supplementary material for: Patient-reported pain and hand function: important determinants of the postoperative results one year after PIP arthroplasty
Source: J Hand Microsurg. 2026 Jan 14;18(2):100421. doi: 10.1016/j.jham.2026.100421 (PMC12874284; doi:10.1016/j.jham.2026.100421)
Supplement: Multimedia component 1 [file mmc1.docx]

| Table S1. Responder analysis |  | |  |  |  |  |  |  |
| --- | --- | --- | --- | --- | --- | --- | --- | --- |
|  |  | **MHQ population** | | **Responder** | | **Non-responder** | | **P-value** |
| **Characteristics** |  | Total (181) | | Included (113) | | Not included (68) | |  |
| Males |  | 45 | 25% | 27 | 24% | 18 | 26% | 0.833 |
| Type of work |  |  |  |  |  |  |  |  |
| Not working (incl. retirement, unable to work) |  | 98 | 54% | 67 | 59% | 31 | 46% | 0.278 |
| Light physical occupation (e.g. office work) |  | 34 | 19% | 20 | 18% | 14 | 21% |  |
| Medium physical occupation (e.g. working in a store) |  | 36 | 20% | 20 | 18% | 16 | 24% |  |
| Heavy physical occupation (e.g. construction work) |  | 13 | 7% | 6 | 5% | 7 | 10% |  |
| Operated on the right hand |  | 90 | 50% | 56 | 50% | 34 | 50% | > 0.99 |
| Age, mean (SD) |  | 61 (10) |  | 63 (9.3) |  | 59 (11) |  | *0.029* |
| Duration of symptoms, median [IQR] |  | 24 [12-60] | | 24 [12-60] | | 24 [12-60] | | 0.941 |
| MHQ baseline subscale hand function, mean (SD) |  | 51 (18) |  | 52 (17) |  | 48 (19) |  | 0.141 |
| MHQ baseline subscale pain, mean (SD) |  | 40 (19) |  | 41 (19) |  | 38 (19) |  | 0.365 |
|  |  |  |  |  |  |  |  |  |
